# Peroxisome Proliferator-Activated Receptor α Activation Induces Hepatic Steatosis, Suggesting an Adverse Effect

**DOI:** 10.1371/journal.pone.0099245

**Published:** 2014-06-13

**Authors:** Fang Yan, Qi Wang, Chao Xu, Mingfeng Cao, Xiaoming Zhou, Tingting Wang, Chunxiao Yu, Fei Jing, Wenbin Chen, Ling Gao, Jiajun Zhao

**Affiliations:** 1 Department of Endocrinology and Metabolism, Shandong Provincial Hospital affiliated to Shandong University, Jinan, Shandong, China; 2 Scientific Center, Shandong Provincial Hospital affiliated to Shandong University, Jinan, Shandong, China; 3 Institute of Endocrinology, Shandong Academy of Clinical Medicine, Jinan, Shandong, China; 4 Institute of Pharmacology, Shandong University, Jinan, Shandong, China; University of Salento, Italy

## Abstract

Non-alcoholic fatty liver disease (NAFLD) is characterized by hepatic triglyceride accumulation, ranging from steatosis to steatohepatitis and cirrhosis. NAFLD is a risk factor for cardiovascular diseases and is associated with metabolic syndrome. Antihyperlipidemic drugs are recommended as part of the treatment for NAFLD patients. Although fibrates activate peroxisome proliferator-activated receptor α (PPARα), leading to the reduction of serum triglyceride levels, the effects of these drugs on NAFLD remain controversial. Clinical studies have reported that PPARα activation does not improve hepatic steatosis. In the present study, we focused on exploring the effect and mechanism of PPARα activation on hepatic triglyceride accumulation and hepatic steatosis. Male C57BL/6J mice, *Pparα*-null mice and HepG2 cells were treated with fenofibrate, one of the most commonly used fibrate drugs. Both low and high doses of fenofibrate were administered. Hepatic steatosis was detected through oil red O staining and electron microscopy. Notably, in fenofibrate-treated mice, the serum triglyceride levels were reduced and the hepatic triglyceride content was increased in a dose-dependent manner. Oil red O staining of liver sections demonstrated that fenofibrate-fed mice accumulated abundant neutral lipids. Fenofibrate also increased the intracellular triglyceride content in HepG2 cells. The expression of sterol regulatory element-binding protein 1c (SREBP-1c) and the key genes associated with lipogenesis were increased in fenofibrate-treated mouse livers and HepG2 cells in a dose-dependent manner. However, the effect was strongly impaired in *Pparα*-null mice treated with fenofibrate. Fenofibrate treatment induced mature SREBP-1c expression via the direct binding of PPARα to the DR1 motif of the *SREBP-1c* gene. Taken together, these findings indicate the molecular mechanism by which PPARα activation increases liver triglyceride accumulation and suggest an adverse effect of fibrates on the pathogenesis of hepatic steatosis.

## Introduction

Non-alcoholic fatty liver disease (NAFLD) represents a spectrum of diseases ranging from hepatic steatosis to steatohepatitis and cirrhosis. The hallmark of NAFLD is excess triglyceride accumulation within hepatocytes [1]. NAFLD is the most common liver disease in Western countries; approximately one third of all Western populations are affected, and the prevalence of these diseases continues to progressively increase [2]. Emerging evidence suggests that NAFLD is the hepatic manifestation of metabolic syndrome and is a risk factor for cardiovascular diseases [3]. Antihyperlipidemic drugs are recommended as part of the treatment for patients with NAFLD [4]. Fibrates are synthetic ligands of peroxisome proliferator-activated receptor α (PPARα), and they serve as first-line drugs for reducing serum triglyceride levels [5]. The lipid-lowering action of fibrates in the blood is mediated through the activation of PPARα and lipoprotein lipase and the suppression of apolipoprotein C-III, among other proteins. [6]–[9]. Theoretically, fibrates might be beneficial for the treatment of NAFLD.

However, no definitive conclusion on the efficacy of PPARα agonists in the treatment of NAFLD can be drawn based on the available clinical data [10]. Some studies have suggested that PPARα activation might have protective and therapeutic effects against NAFLD [10], while others have reported contrasting findings. Fenofibrate, one of the most commonly used fibrates, was reported to exert no beneficial effect on liver steatosis, as assessed using MRI [11]. In 16 patients with biopsy-confirmed NAFLD, 48 weeks of therapy with fenofibrate did not reveal any significant change in the grade of steatosis, lobular inflammation, fibrosis, or the NAFLD activity score when determined by liver histology [12]. Another study investigated liver biopsies before and after 12 months of clofibrate treatment and revealed no improvement in the histological grade of steatosis, inflammation, or fibrosis [13]. We conducted preliminary experiments exploring the effect of fenofibrate as a monotherapy on NAFLD in several patients. Notably, MRI did not reveal any significant change in the steatosis score (data not shown). Interestingly, experiments with mice have shown that fenofibrate can increase hepatic triglyceride synthesis [14]. However, the detection of liver steatosis was not discussed in previous studies. Thus, there is a great need to determine the effect of fibrates on hepatic steatosis as well as the mechanism underlying its effects.

Based on the evidence obtained from previous studies, we hypothesized that PPARα activation induces, rather than improves, hepatic steatosis. In the present study, we showed that fenofibrate treatment increased hepatic steatosis and the liver triglyceride content through the up-regulation of mature SREBP-1c (a key lipogenic transcription factor) expression via the direct binding of PPARα to the DR1 motif of the *SREBP-1c* gene [15]. These findings indicate an adverse effect of fibrates on the pathogenesis of hepatic steatosis. Therefore, the proper use of fibrates should be considered, particularly for the treatment of fatty liver disease.

## Materials and Methods

### Ethics statement

The use of animals in this study was in compliance with the relevant federal guidelines and institutional policies, and the animal protocol was approved by the Animal Care and Use Committee of Shandong Provincial Hospital affiliated with Shandong University (approval number: No. 2013-087). All surgical procedures were performed under sodium pentobarbital anesthesia, and all efforts were made to minimize suffering.

### Animal experiments

All animals were housed in a temperature-controlled room (22–23°C) under a 12-h light/12-h dark cycle and given free access to food and water.

Eight-week-old C57BL/6J male mice (Vital River Company, Beijing, China) were used. The mice were randomly divided into three groups (6 each), including the control group. Fenofibrate (Sigma-Aldrich, St. Louis, MO) suspended in a 1% carboxymethylcellulose solution of gum Arabic was administered through daily gavage for 10 days at a dose of 0.04 g/kg/day or 0.5 g/kg/day. Animals receiving vehicle alone were used as controls.

*Pparα*-null mice (*Pparα^−/−^*) on a 129S (129S4/SvJae-*Ppara*<tm1Gonz>/N) background have been previously described [16] and were kindly provided by Prof. Gonzalez FJ (National Cancer Research, National Institutes of Health, USA). Eight-week-old male *Pparα^−/−^* mice and their wild-type (*Pparα^+/+^*) counterparts (the Jackson Laboratory, Bar Harbor, ME, USA, stock number 2448) were fed fenofibrate at a dose of 0.5 g/kg/day or vehicle through daily gavage for 10 days (6 mice per group).

The mice were fasted for 6 h and then euthanized using pentobarbital sodium. Serum was collected immediately prior to sacrificing the mice. The livers were immediately harvested and frozen in liquid nitrogen for further experiments. Part of the liver was frozen in optimal cutting temperature compound (OCT) embedding medium in liquid nitrogen.

### Human hepatic cell lines and mouse primary hepatocyte isolation and culture

The HepG2 human hepatocellular carcinoma cell line was obtained from the Type Culture Collection of the Chinese Academy of Sciences, Shanghai, China. HepG2 cells were routinely maintained in MEM/EBSS (HyClone, Logan, UT, USA) supplemented with 10% fetal bovine serum (Gibco BRL, Gaithersburg, MD, USA), 100 U/mL penicillin, and 100 µg/mL streptomycin at 37°C in a humidified atmosphere of 5% CO_2_.

Hepatocytes were isolated from C57BL/6J male mice using the two-step collagenase perfusion protocol [17]. Briefly, mice were anesthetized with sodium pentobarbital (30 mg/kg intraperitoneally), and the portal vein was cannulated under aseptic conditions. The liver was perfused with 0.9% saline containing 0.5 mM EDTA and low-glucose DMEM (HyClone) containing 100 CDU/ml collagenase type IV (Sigma-Aldrich). The isolated mouse hepatocytes were then cultured at 80%–90% confluence in DMEM media (HyClone) containing 10% FBS in rat-tail collagen type I (Sigma-Aldrich) coated plates. The cells were then incubated overnight at 37°C in a humidified atmosphere of 5% CO_2_.

When treated with fenofibrate, the cells were washed twice with PBS and then starved in serum-free medium overnight before treatment. The cells were cultured in serum-free medium during treatment.

### Plasmid construction and transfection

pSV-SPORT plasmids encoding a dominant negative mutant of rat SREBP-1c (DN-SREBP-1c) were purchased from Addgene (Addgene plasmid 8885, https://www.addgene.org/). The luciferase reporter construct containing the wild-type human SREBP-1c promoter, from -1564 to +1, has been previously described [15] and was kindly provided by Dr. Marta Casado (IBV-CSIC, Valencia, Spain). Transfection was performed using Lipofectamine 2000 (Invitrogen, Carlsbad, CA).

### Gene silencing using siRNA

Small interfering RNAs (siRNAs) targeting the human *PPARα* gene were designed at BioSune (Shanghai, China). The sequences were as follows: sense, 5' GGAGCAUUGAACAUCGAAUTT 3'; antisense, 5' AUUCGAUGUUCAAUGCUCCTT 3'.

### Dual luciferase activity assays

Cells were cotransfected with 0.4 µg of luciferase reporter plasmid and 20 ng of *Renilla* luciferase plasmid pRL-SV40 (Promega, Madison, WI) as an internal control. Ten hours after transfection, the medium was changed, and the cells were allowed to recover for an additional 8 h. The cells were treated with fenofibrate in serum-free medium for 24 h. The cells were then harvested, and luciferase activity was measured using a dual-luciferase reporter assay system (Promega). Data represent the amount of firefly luciferase activity, normalized to that of *Renilla* luciferase activity.

### RNA isolation and quantitative RT-PCR

Total RNA from cells and mouse liver tissue was isolated using TRIzol reagent (Takara, Tokyo, Japan) following the manufacturer's instructions. The RT reaction was performed using 1 µg of total RNA. Real-time PCR was performed with a Light Cycler 480 (Roche Applied Science, Indianapolis, IN) [18]. The PCR primers are shown in Table 1 (for human) and Table 2 (for mice). *β*-actin was employed as an endogenous control for normalization.

**Table 1:** Primers used for the analysis of mRNA expression levels in humans.

Gene	NM	Product (bp)	Forward primer	Reverse primer
**Srebp1c**	NM_001005291.2	80	GGAGGGGTAGGGCCAACGGCCT	CATGTCTTCGAAAGTGCAATCC
**PPARα**	NM_001001928.2	304	AAGGGCTTCTTTCGGCGAAC	TGACCTTGTTCATGTTGAAGTTCTTCA
**SCD1**	NM_005063.4	281	CCTCTACTTGGAAGACGACATTCG	GCAGCCGAGCTTTGTAAGAGC
**FASN**	NM_004104.4	159	CGGAAACTGCAGGAGCTGTC	CACGGAGTTGAGCCGCAT
**ACC**	NM_198834.1	253	GAATGTTTGGGGATATTTCAG	TTCTGCTATCAGTCTGTCCAG
**Cpt1α**	NM_001031847.2	133	CCTCCAGTTGGCTTATCGTG	TTCTTCGTCTGGCTGGACAT
**Srebp1a**	NM_001005291.2	135	ATGGACGAGCCACCCTTC	GCCAGGGAAGTCACTGTCTTG
**FABP1**	NM_002080.2	142	ATCCCACGGGAGTGGACCCG	CGCACAGCCCAGGCATCCTT
**ApoB**	NM_000384.2	107	CAACCCTGAGGGCAAAGCCTTGCTG	CCTGCTTCCCTTCTGGAATGGCC
**DGAT2**	NM_001253891.1	215	TGGGGGCTGGTGCCCTACTC	AATTGGCCCCGAAGGCTGGA
**GPAT1**	NM_001244949.1	154	AACCCCAGTATCCCGTCTTT	CAGTCACATTGGTGGCAAAC
**β-actin**	NM_001101.3	104	ACAGAGCCTCGCCTTTGCCG	ACATGCCGGAGCCGTTGTCG

**Table 2:** Primers used for the analysis of mRNA expression levels in mice.

Gene	NM	Product (bp)	Forward primer	Reverse primer
**Srebp1c**	NM_011480.3	113	GCGCTACCGGTCTTCTATCA	GGATGTAGTCGATGGCCTTG
**PPARα**	NM_001001928.2	304	AAGGGCTTCTTTCGGCGAAC	TGACCTTGTTCATGTTGAAGTTCTTCA
**SCD1**	NM_009127.4	242	AAGATATTCACGACCCCACC	CAGCCGTGCCTTGTAAGTTC
**FASN**	NM_007988.3	234	GTCCTGGGAGGAATGTAAACAG	CGGATCACCTTCTTGAGAGC
**ACC**	NM_133360.2	235	GCTTATTGATCAGTTATGTGGCC	CTGCAGGTTCTCAATGCAAA
**Cpt1α**	NM_013495.2	100	TTGGGCCGGTTGCTGAT	GTCTCAGGGCTAGAGAACTTGGAA
**Srebp1a**	NM_011480.3	69	GGCCGAGATGTGCGAACT	TTGTTGATGAGCTGGAGCATGT
**FABP1**	NM_017399.4	74	TCAAGCTGGAAGGTGACAATAA	GTCTCCATTGAGTTCAGTCACG
**ApoB**	NM_009693.2	121	AAACATGCAGAGCTACTTTGGAG	TTTAGGATCACTTCCTGGTCAAA
**DGAT2**	NM_026384.3	66	AGAACCGCAAAGGCTTTGTG	AGGAATAAGTGGGAACCAGATCAG
**GPAT1**	NM_008149.3	67	CAACACCATCCCCGACATC	GTGACCTTCGATTATGCGATCA
**β-actin**	NM 007393.3	101	ACCCCAGCCATGTACGTAGC	GTGTGGGTGACCCCGTCTC

### Quantification of the triglyceride content

The triglyceride content was measured using a colorimetric assay (Applygen Technologies Inc., Beijing, China) as previously reported [19]–[21]. Briefly, the liver homogenate was prepared after homogenizing the tissue (∼50 mg) in 1 ml of standard diluent. The samples were centrifuged at 2000 g for 10 min, and the supernatant was collected. The absorbance at 550 nm is proportional to the concentration of triglycerides of each sample. All samples were determined in duplicate, and the triglyceride values were expressed as mmol of triglycerides/g of protein (for liver) or mmol/L (for serum).

### Oil red O staining

The cultured cells were washed twice with PBS and then fixed with 4% paraformaldehyde for 15 min and stained for 15 minutes in a freshly diluted oil red O (Sigma-Aldrich) solution. The cells were counterstained with hematoxylin for 10 sec. To evaluate hepatic lipid accumulation, sections of the liver (10 µm) frozen in OCT embedding medium were stained with oil red O for 10 minutes and then washed and counterstained with hematoxylin for 20 seconds. Representative photomicrographs were captured using a system incorporated into the microscope (Axiovert 100 M Zeiss, Zeppelinstrasse, Germany).

### Electron microscopy

Cells were first fixed with 3.5% (v/v) glutaraldehyde in phosphate buffer (pH 7.2) at room temperature overnight and then post-fixed using 1% osmic acid, dehydrated through an ethanol series, and embedded in Spurr's low-viscosity resin. Transverse ultrathin were prepared and contrasted with saturated uranyl acetate and lead citrate. Microphotographs were taken using a Jeol 1200X electron microscope (Jeol System Co., Akishima, Tokyo, Japan).

### Nuclear and cytoplasmic protein extraction and Western blotting

Nuclear and cytoplasmic extracts from cultured hepatocytes and mouse livers were prepared using the NE-PER nuclear and cytoplasmic extraction reagent kit (Pierce Biotechnology, Rockford, IL) according to the manufacturer's instructions. Protein content was determined using a BCA Protein Assay Kit (Shenergy Biocolor Bioscience & Technology Company, Shanghai, China). Protein from nuclear extracts (40–60 µg) or cytoplasmic extracts (60–80 µg) was electrotransferred onto a polyvinylidene fluoride membrane (Millipore, Billerica, MA, USA), and after incubation in 5% BSA for one hour, the blots were probed with the following antibodies at the dilution indicated: SREBP-1 (1∶200; Santa Cruz) and PPARα (1∶1000; Millipore) at 4°C for the entire night. Mouse anti-LMB1 antibody and anti-GAPDH antibody were obtained from Cwbiotech (Beijing, China) and were used to target endogenous control proteins in the nuclear and cytosolic fractions, respectively. After incubation with the appropriate secondary antibodies conjugated to horseradish peroxidase (HRP) (Amersham, Little Chalfont Bucks, UK) at 1∶5,000 for one hour at room temperature, the membranes were visualized using a HyGLO HRP detection kit (Denville, NJ, USA). Quantification of Western blots was performed using ImageJ software (developed at the National Institutes of Health, Bethesda, Maryland).

### Immunofluorescence

Cells attached to coverslips were washed with PBS and fixed in 4% paraformaldehyde. The cells were then blocked with 10% normal goat serum for 30 minutes and then incubated with primary antibodies (rabbit anti-SREBP1 or mouse anti-PPARα, both 1∶100 dilution) overnight at 4°C, followed by a 1 h incubation at room temperature with fluorescein isothiocyanate (FITC)-conjugated goat anti-rabbit IgG (1∶100 dilutions). The nuclei of hepatocytes were stained with DAPI. Specimens were imaged under a confocal fluorescence microscope (Axiovert 100 M Zeiss, Zeppelinstrasse, Germany).

### Statistical analysis

The data were analyzed using SPSS 17.0 and are expressed as the mean ± standard deviation. Differences between two groups were compared using an unpaired Student's *t*-test. ANOVA was used to compare the means of multiple groups. All of the calculated *P* values are two-sided. Differences were considered significant at *P*<0.05.

## Results

### Liver triglyceride homeostasis was disrupted through pharmacological treatment with fenofibrate

**Figure 1:**
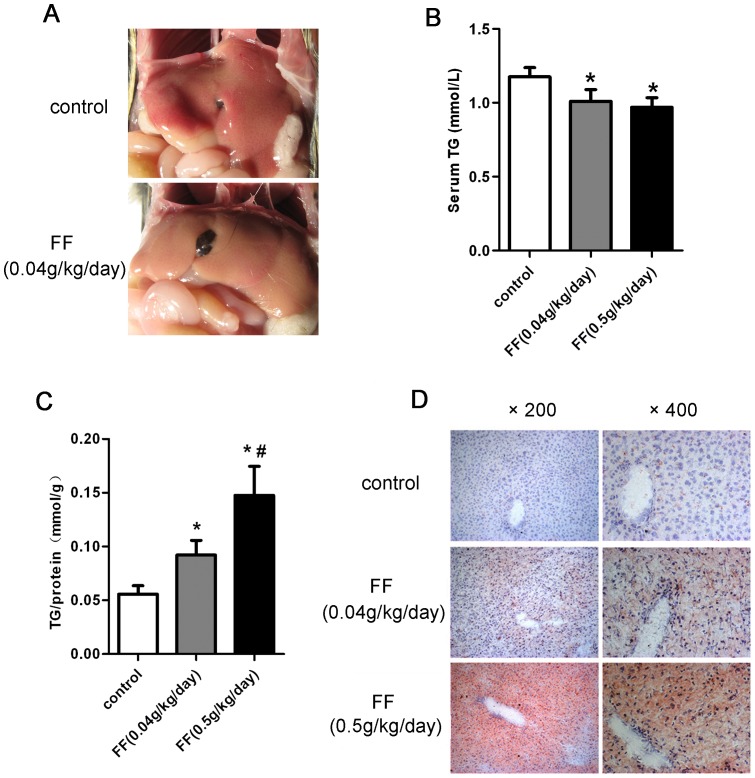
Activation of PPARα through fenofibrate promoted liver triglyceride accumulation *in vivo*. (A-D) Eight-week-old male C57BL/6 mice were orally treated with or without fenofibrate at the indicated dosage (n = 6 per group) for 10 days: (A) Representative gross morphology of the mouse livers. (B) Serum triglyceride (TG) levels and (C) liver TG contents were determined using a commercial kit. All samples were evaluated in duplicate, and the liver TG values are expressed in mmol of triglycerides/g of protein. (D) Representative images of oil red O staining of liver sections for the detection of neutral lipids. The data are presented as the mean ± SD. * *P*<0.05 *versus* the control group. # *P*<0.05 *versus* the fenofibrate 0.04 g/kg/day group.

The PPARα agonist fenofibrate has been commonly used in humans and animals in previous studies [22]. As shown in Table 3, fenofibrate treatment induced weight loss in the mice. However, glutamic-pyruvic transaminase (ALT), glutamic-oxaloacetic transaminase (AST), and the ratio of liver weight to body weight were increased in mice treated with high doses of fenofibrate compared with the control mice. The liver of fenofibrate-fed mice was pale in color, suggesting increased lipid storage (**Fig. 1A**). To determine the role of PPARα in triglyceride metabolism, the serum and hepatic triglyceride contents were measured. Consistent with previous studies [23], the serum triglyceride levels were significantly reduced in response to fenofibrate treatment (**Fig. 1B**), whereas the hepatic triglyceride contents increased in a dose-dependent manner after fenofibrate gavage (**Fig. 1C**). Oil red O staining of liver sections from fenofibrate-fed mice showed accumulated neutral lipids (**Fig. 1D**), which was consistent with the gross morphological appearance. These results indicated that fenofibrate treatment induced triglyceride accumulation in hepatocytes, which leads to liver steatosis.

**Table 3:** General characteristics, plasma, and hepatic metabolite levels.

	Control	Fenofibrate (0.04 g/kg/day)	Fenofibrate (0.5 g/kg/day)
**Body weight change (%)**	2.25±0.53	−0.61±0.40	−9.75±1.25*
**Liver weight (% body weight)**	4.06±0.36	4.81±0.56	7.32±0.46*
**ALT (IU/L)**	34±4.57	32±2.53	148±15.01*
**AST (IU/L)**	127±17.32	147±3.01	229±19.37*

Values are given as the mean ± SD. for n = 6; *, p<0.05 *vs.* control mice.

**Figure 2:**
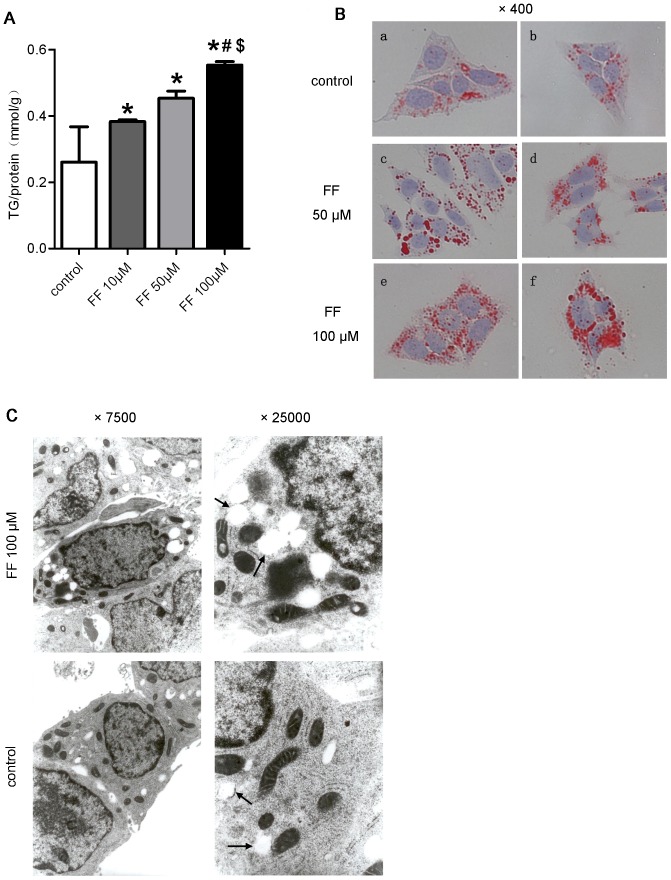
Fenofibrate promoted triglyceride accumulation in HepG2 cells. (A-C) HepG2 cells were cultured in serum-starved medium with or without fenofibrate for 48 h: (A) cellular TG contents, (B) oil red O staining, and (C) electron micrographs showing the increased accumulation of lipid droplets (arrows) in the cytoplasm. The data are presented as the means ± SD. * *P*<0.05 *versus* the control group. # *P*<0.05 *versus* the fenofibrate 10 µM group. $ *P*<0.05 *versus* the fenofibrate 50 µM group.

The effect of PPARα activation was confirmed in HepG2 cells treated with fenofibrate. Cellular triglyceride contents increased in a concentration-dependent manner in response to fenofibrate treatment (**Fig. 2A**). In addition, oil red O staining demonstrated the accumulation of neutral lipids in fenofibrate-treated cells (**Fig. 2B**). Furthermore, the electron microscopic analysis of HepG2 cells revealed only a few lipid droplets in the control cells. Fenofibrate treatment, however, induced the production of a large number of medium-to-large lipid droplets (**Fig. 2C**). These data confirmed that fenofibrate increased the intracellular triglyceride content.

### PPARα activation induced SREBP-1 gene expression *in vivo* and *in vitro*

**Figure 3:**
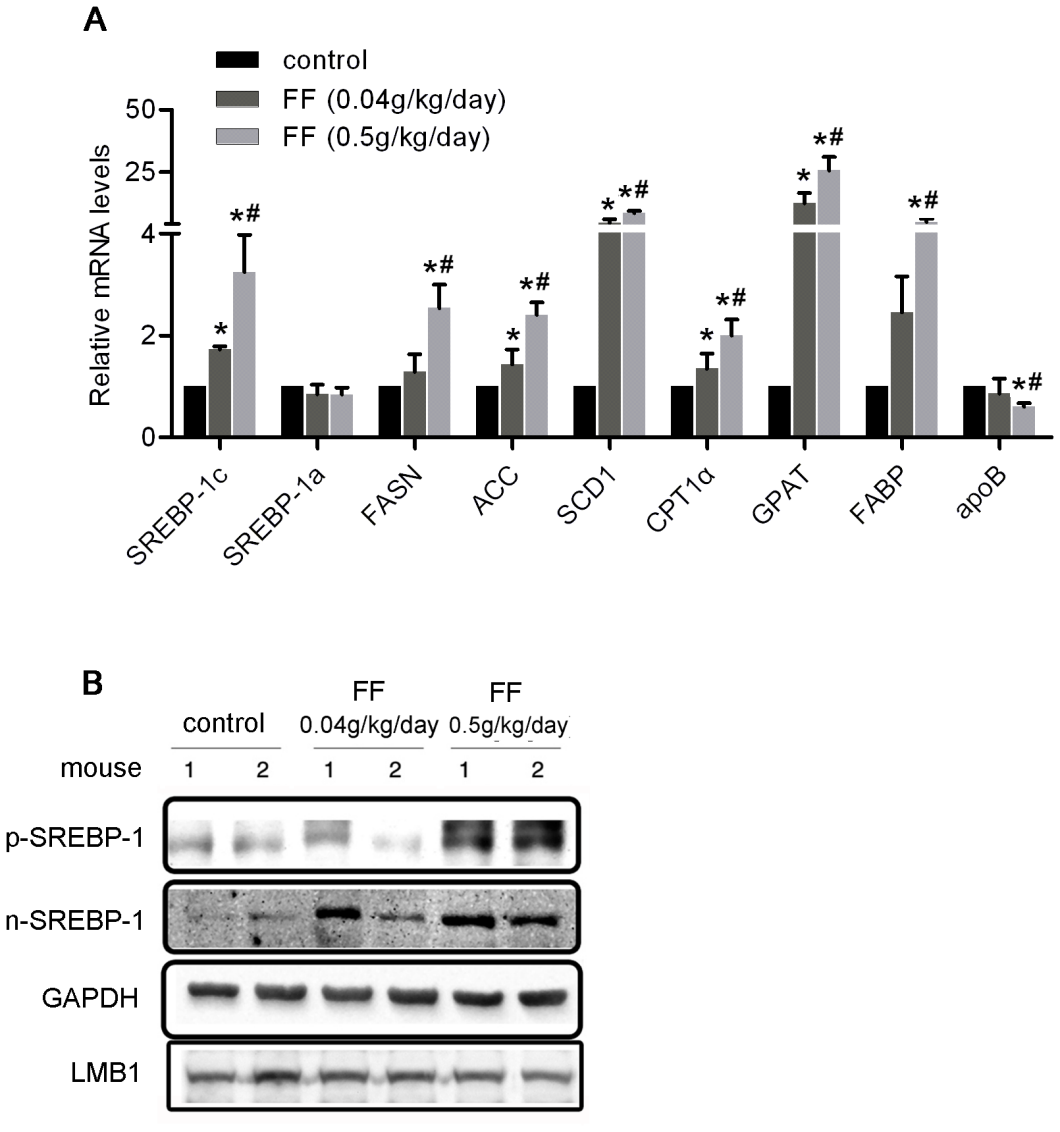
Pharmacological activation of PPARα by fenofibrate induced the expression of SREBP-1c. (A-B) Eight-week-old male C57BL/6 mice were orally treated with or without fenofibrate at the indicated dosage (n = 6 per group) for 10 days: liver mRNA levels of *SREBP-1c* and related TG metabolism genes were determined using real-time PCR and normalized to *β-actin*. The values are reported as the fold-change relative to control mice, * *P*<0.05 *versus* the control group, # *P*<0.05 *versus* the fenofibrate 0.04 g/kg/day group and (B) the p-SREBP-1 and n-SREBP-1 proteins in the cytosolic and nuclear fractions, respectively, in the liver were detected through Western blotting. GAPDH and LMB1 were used as marker proteins for the cytosolic and nuclear fractions, respectively. The “1” and “2” represented different samples from different mouse in each group.

To elucidate the mechanisms underlying the influence of fenofibrate on the triglyceride content in the liver, we examined the expression of the genes involved in triglyceride metabolism. As shown in **Fig. 3A**, the expression of Cpt1α, which is directly regulated through PPARα, was increased upon fenofibrate treatment, indicating the activation of PPARα. Subsequently, the expression of SREBP-1c, a key regulatory molecule involved in lipogenesis, was significantly increased in the livers of fenofibrate-treated mice, and SREBP-1a expression was not significantly affected. Expression of the key genes associated with lipogenesis including ACC (acetyl-CoA carboxylase), FASN (fatty acid synthase), SCD1 (stearoyl-CoA desaturase 1), and GPAT (glycerol phosphate acyltransferase), was also increased in the fenofibrate-treated mouse livers. Interestingly, the transcription level of these genes in response to fenofibrate treatment showed a dose-dependent increase in parallel with the level of SREBP-1c expression. The expression of fatty acid-binding protein 1 (FABP1), which regulates the cellular uptake of long-chain fatty acids, was enhanced, and the expression of apoB, which regulates triglyceride exportation from the liver, was reduced in fenofibrate-treated mouse livers. These findings are consistent with the results of a previous study [24].

To further evaluate whether the expression of SREBP-1c was induced during the lipogenesis resulting from fenofibrate treatment, we examined liver extracts using Western blotting. Notably, prominent increases in the precursor and mature forms of SREBP-1 proteins were observed in fenofibrate-treated mouse livers (**Fig. 3B**).

**Figure 4:**
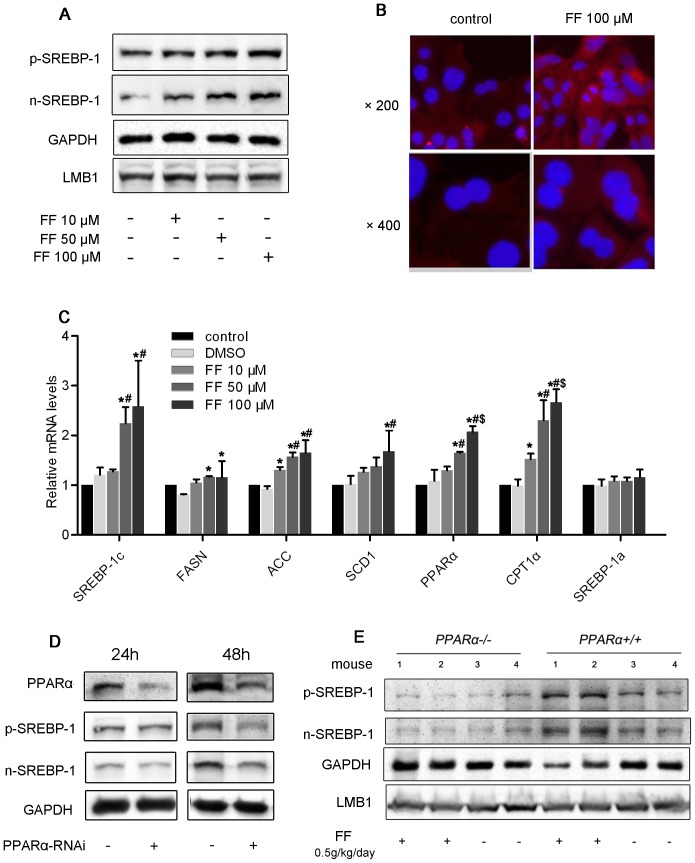
Fenofibrate induced the expression of SREBP-1c in HepG2 cells. (A) HepG2 cells were cultured in serum-starved medium with or without fenofibrate for 48 h. Protein expression was analyzed using Western blotting of nuclear and cytosolic fractions. The results are representative of 3 independent experiments. (B) Visualization of SREBP-1 protein expression in cells through immunofluorescence staining. Primary hepatocytes were cultured in serum-free medium and stimulated with fenofibrate for 48 h. SREBP-1 protein was labeled with a fluorescent antibody (red), and nuclei were stained with DAPI (blue). Original magnification: 400×. (C) HepG2 cells were cultured in serum-free medium with or without fenofibrate for 24 h. The mRNA expression of *SREBP-1c* and related genes after 24 h was determined using real-time PCR and normalized to *β-actin*. The values are reported as the fold-change relative to the control group. The data are expressed as the mean ± SD. * *P*<0.05 *versus* the DMSO group. # *P*<0.05 *versus* the fenofibrate 10 µM group. $ *P*<0.05 *versus* the fenofibrate 50 µM group. (D) HepG2 cells were transfected with PPARα-RNAi or control-RNAi for 24 or 48 h, and SREBP-1 protein expression was analyzed by Western blotting. (E) Eight-week-old male *Pparα^−/−^* mice and *Pparα^+/+^* mice were orally treated with or without fenofibrate at a dosage of 0.5 g/kg/day (n = 6 per group) for 10 days: p-SREBP-1 and n-SREBP-1 protein expression was detected by Western blotting. The “1”, “2”, “3” and “4” represented different samples from different mouse in each group.

To reconfirm the effect of PPARα activation on the induction of SREBP-1 gene expression, we treated HepG2 cells with fenofibrate. Notably, fenofibrate increased the expression of SREBP-1c protein in a dose-dependent manner in HepG2 cells treated for 48 h (**Fig. 4A**). Immunofluorescence analysis of mouse primary hepatocytes revealed strong SREBP-1 staining in the nucleus and cytoplasm of these cells (**Fig. 4B**). Fenofibrate incubation increased SREBP-1 expression in the cytoplasm and promoted the translocation of this gene to the nuclei. In addition, real-time PCR analysis revealed prominent elevations in SREBP-1c and its downstream molecules, such as FASN, ACC, and SCD1, while SREBP-1a showed no change (**Fig. 4C**). Interestingly, the expression of both the precursor and mature forms of SREBP-1 correspondingly decreased when PPARα was silenced by siRNA in HepG2 cells (**Fig. 4D**).

To determine whether the induction of SREBP-1 expression observed in fenofibrate-treated mice is dependent on PPARα activation, we used *Pparα^−/−^* mice. As shown in **Fig. 4E**, fenofibrate gavaging increased SREBP-1 protein expression in *Pparα^+/+^* mouse livers, whereas this effect was abolished in the PPARα^−/−^ mice.

### Direct regulation of SREBP-1c by PPARα and SREBP-1c was indispensable in PPARα-induced liver triglyceride accumulation

**Figure 5:**
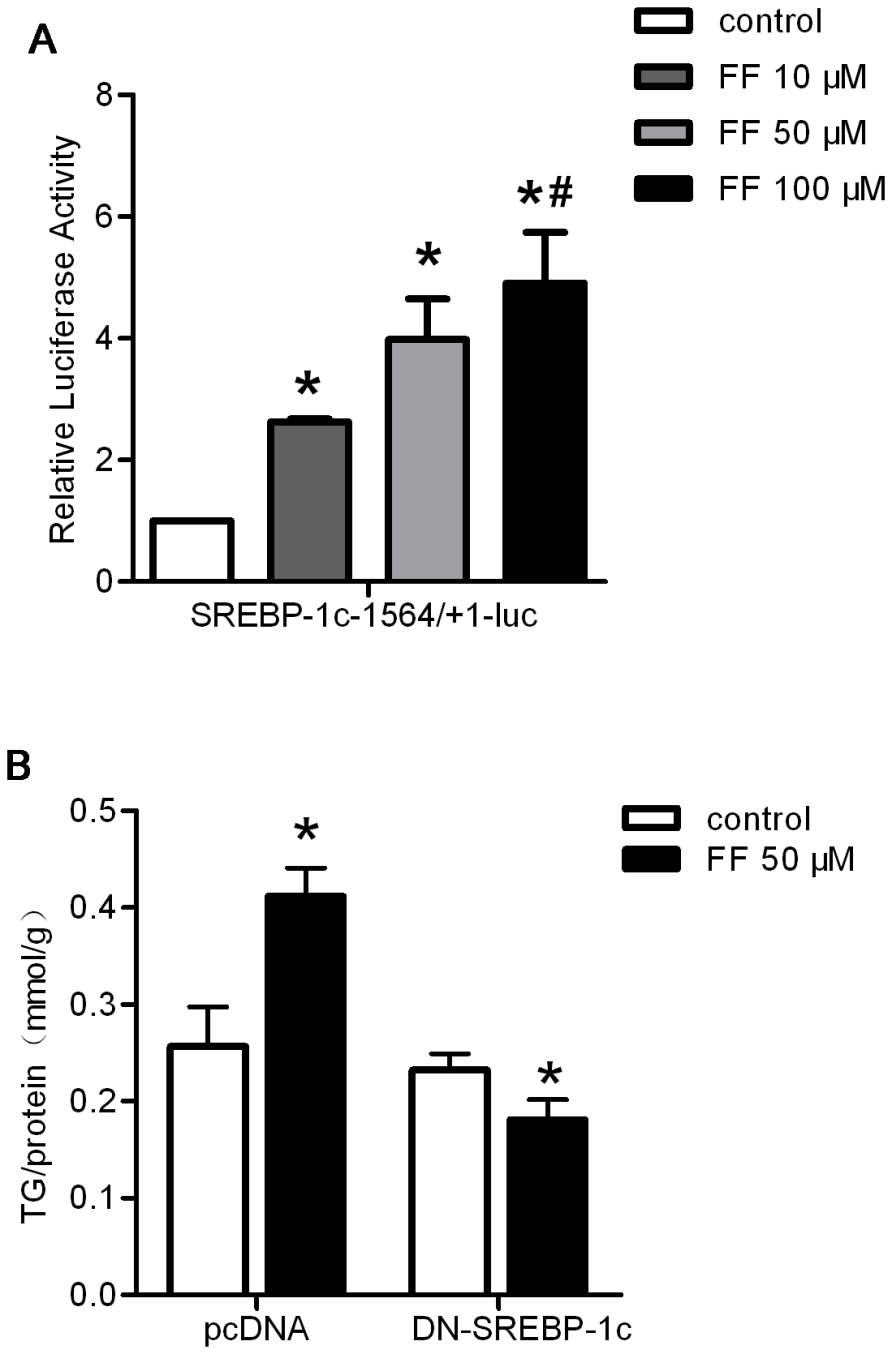
Direct regulation of SREBP-1c through PPARα was indispensable in PPARα-induced liver triglyceride accumulation. (A) PPARα-dependent regulation of the SREBP-1c promoter was analyzed through luciferase assays using the human SREBP-1c-1564/+1-luc construct in the presence of the indicated amounts of fenofibrate for 24 h. The activities of the constructs transfected into HepG2 cells under the indicated dosages are shown. The results represent relative Firefly/Renilla luciferase activities compared with the wild-type construct under basal conditions as the reference value. (B) HepG2 cells were transfected with the empty plasmid pcDNA3.1 or DN-SREBP-1c and subsequently treated with or without 50 µM fenofibrate in serum-free medium for an additional 48 hours. The cells were collected and assayed to determine the intracellular TG content. The content was normalized to the total protein from the same group. The data were calculated from 3 independent experiments and are expressed as the mean ± SD. * *P*<0.05 *versus* the control group. # *P*<0.05 *versus* the fenofibrate 10 µM group.

Studies have reported that SREBP-1c expression is reduced in *Pparα^−/−^* mice compared with wild-type mice [14], [25], [26]. Indeed, PPARα agonists enhance the activity of the *Srebp-1c* promoter through direct binding with the DR1 motif [15]. Using the full-length SREBP-1c promoter (1564/+1)-driven luciferase construct, we observed that luciferase activity was significantly increased by fenofibrate treatment in a dose-dependent manner, indicating that SREBP-1c expression is directly regulated through PPARα (**Fig. 5A**).

To determine the indispensable role of SREBP-1c in PPARα-induced hepatic triglyceride accumulation, we used a plasmid encoding DN-SREBP-1c. DN-SREBP-1c contains a tyrosine 320 to arginine mutation on the truncated nuclear form of rat SREBP-1c (1-403R), which disrupts the binding of SREBP-1c to the SRE motif [27]. Interestingly, DN-SREBP-1c completely inhibited the fenofibrate-mediated increase in the hepatic triglyceride content (**Fig. 5B**). These results suggest that SREBP-1c is necessary for PPARα-induced liver lipid accumulation.

## Discussion

Using a series of *in vivo* and *in vitro* experiments, we confirmed that PPARα activation through fenofibrate increased liver triglyceride synthesis, leading to hepatic steatosis. The effect of fenofibrate was observed at both low and high doses. Fenofibrate treatment induced mature SREBP-1c expression via the direct binding of PPARα to the DR1 motif of the *SREBP-1c* gene, which up-regulates the expression of the key genes associated with lipogenesis. These findings suggest a molecular mechanism that underlies specific clinical findings, showing that fibrates cannot improve hepatic steatosis in patients with NAFLD [11]–[13]. Based on these results and previous clinical findings, the efficacy of fibrates, particularly in the treatment of fatty liver disease, should be re-evaluated, indicating a need for large prospective studies and a full assessment of liver histology.

Fenofibrate is available for oral administration at a daily dose of 200–300 mg (4–5 mg/kg/day for 60 kg) in adult patients in the clinic [28], and a previous study reported that the blood concentration reached 30 µM after fenofibrate treatment at 200 mg daily for 7 days [29]. Based on these data, we adopted 0.04 g/kg daily as a low *in vivo* (therapeutic dose) dosage and 0.5 g/kg daily as a high *in vivo* (overdose) dosage for treating mice; we also used 50 and 100 µM concentrations *in vitro* to stimulate hepatocytes. The results showed that fenofibrate increased the expression of the genes involved in triglyceride synthesis and fatty acid uptake, transport, synthesis, and β-oxidation, increasing the triglyceride content in the liver, which is consistent with previous studies [23]. The induction of weight loss by a high dose of fenofibrate was observed in the present and previous studies [14]. Elevated plasma ALT and AST levels were also observed. However, it appears unlikely that the induction of liver steatosis by fenofibrate was the result of liver damage. Indeed, treatment with the low dose of fenofibrate, in which ALT and AST remained normal, also induced liver triglyceride accumulation, indicating a direct role of fenofibrate in liver steatosis. In addition, Nakajima T *et al* also showed remarkable differences in bezafibrate action on PPARα activation and reactive oxygen species generation between conventional experimental high doses and clinically relevant low doses in wild-type mice [30]. Thus, despite the use of a different molecule, these findings support the differences observed in the present study.

Some clinical studies have assessed the effects of fenofibrate on biochemical and imaging surrogates of NAFLD [11]–[13]. Indeed, recent preclinical studies have strongly suggested that PPARα activation increases liver lipid synthesis. Treatment with a PPARα agonist promotes ^3^H_2_O incorporation into hepatic lipids in wild-type mice but not in *Pparα^−/−^* mice [31]. Additionally, fenofibrate-treated mice show strong acetyl-CoA incorporation into hepatic fatty acids [14]. The normal circadian rhythms of hepatic lipogenic FASN and ACC expression are disturbed in *Pparα^−/−^* mice [25]. Moreover, studies have reported that SREBP-1c mRNA levels are decreased in *Pparα^−/−^* mice compared with wild-type mice, suggesting the PPARα-dependent induction of hepatic fatty acid synthesis and SREBP-1c activation [14], [25], [26]. These findings are consistent with the results of the present study, which showed that PPARα activation induced hepatic triglyceride accumulation through the up-regulation of mature SREBP-1c expression. Notably, compared with previous studies [32], we administered both a therapeutic dose and an overdose of fenofibrate. Moreover, we focused on the effect of fenofibrate on hepatic steatosis, while previous studies did not present similar results. Morphological observations and oil red O staining were used to examine liver steatosis in mice. The effects of fenofibrate on liver lipid accumulation were reconfirmed using electron microscopy.

These findings suggest a direct regulatory effect of PPARα on SREBP-1c. A PPARα response element (DR1) in the promoter of the human SREBP-1 gene has been identified and is involved in PPARα protein binding [33], [34]. Using the dual-luciferase reporter assay system, we demonstrated that fenofibrate treatment enhanced the activity of the human SREBP-1c promoter in a dose-dependent manner. Furthermore, we found that SREBP-1c expression was reduced after the HepG2 cells were treated with PPARα siRNA. Therefore, it is reasonable to conclude that the increased levels of SREBP-1c mRNA and mature protein following PPARα activation were induced by fenofibrate treatment. Although a DR1 motif has not been found in the mouse SREBP-1 promoter [31], the induction of SREBP-1 mRNA expression observed in fenofibrate-treated mice could be due to different molecular mechanisms, which require further study: 1. A PPARα binding site other than DR1 may exist on the mouse SREBP-1c promoter. 2. PPARα exerts an indirect regulatory effect on SREBP-1c in mice. In the present study, the requirement of PPARα for the induction of SREBP-1 was tested in a *Pparα^−/−^* mouse model. The up-regulation of SREBP-1 expression was observed in fenofibrate-treated *Pparα^+/+^* mice, and this effect was strongly impaired in *Pparα^−/−^* mice. The results indicate that the induction of SREBP-1 expression observed in fenofibrate-treated mice is dependent on PPARα activation, similar to the changes observed in other studies [25], [26], [35], [36]. Fibrates also stimulate the β-oxidation of fatty acids, leading to fatty acid depletion, which increases SREBP-1c expression [15]. Notably, PPARα-induced SREBP-1c expression might not occur secondary to fatty acid depletion because treatment with etomoxir, an inhibitor of fatty acid oxidation, does not abolish the effect of WY 14,643 (a PPARα agonist) on the incorporation of ^3^H_2_O into fatty acids [25], [26]. Interestingly, a DR1 element has been found in the promoter region of other lipogenic genes regulated by SREBP-1 [37], and they are under the direct control of PPARα. This is helpful for explaining the development of steatosis observed in fenofibrate-treated mice. The molecular mechanism by which PPARα regulates the mouse SREBP-1c expression remains to be elucidated.

However, some studies have suggested that hepatic triglyceride accumulation might be a protective mechanism through which the toxic effects of free fatty acids are prevented [38] [39]. Moreover, previous studies have demonstrated that PPARα activation might be protective and therapeutic against NAFLD [40]–[42]. This benefit has been associated with improved fatty acid turnover and the anti-inflammatory and anti-oxidant properties of PPARα [43]–[45]. In these studies, the data obtained suggested a role for fenofibrate under conditions of high-fat diet, obesity, insulin resistance, and type 2 diabetes mellitus. In the present study, we administered fenofibrate to normal adult mice, which presented normal serum lipid levels before treatment. The discrepancy between these results and those of previous studies likely reflects the different animal models employed. PPARα activation exerted a synergistic effect on lipid metabolism, which involved accelerated lipid mobilization in white adipose tissue, liver free fatty acid uptake, DNL, fatty acid *β*-oxidation, and exportation. The disease models might perturb this balance, contributing to a different effect of fenofibrate on the hepatic triglyceride content. However, this controversy should be further assessed.

In conclusion, the results of the present study showed that PPARα activation through fenofibrate treatment increased liver triglyceride synthesis, leading to hepatic steatosis. The underlying mechanism involves the induction of mature SREBP-1c expression via the direct regulation of SREBP-1c through PPARα, which further up-regulates the expression of genes associated with lipogenesis. These findings are consistent with the results of previous clinical studies showing that fibrates do not improve hepatic steatosis in patients with NAFLD. Thus, there is a need for large prospective studies and a full assessment of liver histology to reevaluate the efficacy of fibrates, particularly for the treatment of fatty liver disease.

## Acknowledgments

We thank Prof. Gonzalez FJ (National Cancer Research, National Institutes of Health, USA) for providing the *Pparα^−/−^* mice; Prof. Marta Casado (IBV-CSIC, Valencia, Spain) for providing the plasmid and Prof. Xuefeng Xia (Weill Cornell Medical College, USA) for suggestions about the experimental design.
